# Fibroblast Activation Protein-Alpha is a Prognostic Biomarker Associated With Ferroptosis in Stomach Adenocarcinoma

**DOI:** 10.3389/fcell.2022.859999

**Published:** 2022-03-14

**Authors:** Zejian Lyu, Yafang Li, Dandan Zhu, Sifan Wu, Fei Hu, Yu Zhang, Yong Li, Tieying Hou

**Affiliations:** ^1^ The Second School of Clinical Medicine, Southern Medical University, Guangzhou, China; ^2^ Department of Gastrointestinal Surgery, Guangdong Provincial People’s Hospital, Guangdong Academy of Medical Sciences, Guangzhou, China; ^3^ Guangdong Clinical Laboratory Center, Guangdong Provincial People’s Hospital, Guangdong Academy of Medical Sciences, Guangzhou, China; ^4^ School of Medicine, South China University of Technology, Guangzhou, China; ^5^ The First School of Clinical Medicine, Guangdong Medical University, Zhanjiang, China; ^6^ Medical Department, Guangdong Provincial People’s Hospital, Guangdong Academy of Medical Sciences, Guangzhou, China

**Keywords:** FAP, stomach adenocarcinoma, tumor microenvironment, ferroptosis, biomarker

## Abstract

**Background:** The potential role of fibroblast activation protein-alpha (FAP) in modulating the progression and invasion of stomach adenocarcinoma (STAD) has not yet been comprehensively investigated. This study aimed to explore the role of FAP in STAD and the underlying association between FAP and the tumor microenvironment (TME) and ferroptosis.

**Methods:** Overall survival was analyzed to evaluate the prognostic value of FAP based on gene expression data and clinical information on STAD. Associations between FAP expression, clinical parameters, and immune characteristics were comprehensively analyzed. The ferroptosis-related patterns of STAD samples were investigated based on 43 ferroptosis-related genes, and the correlations between these clusters and clinical characteristics were evaluated. The possible biological functions and pathways were explored using gene set enrichment analysis (GSEA).

**Results:** FAP was identified as a novel biomarker that significantly contributed to the poor prognosis of STAD (hazard ratio = 1.270, *P* = 0.013). The elevated level of FAP expression was related to a more advanced tumor stage in STAD. The close relationship between FAP and the TME was validated. Four distinct ferroptosis-related clusters (A–D) were evident. Evaluating ferroptosis-related clusters could illustrate the stages of STAD and patient prognosis. Cluster C displayed the lowest FAP expression and a better prognosis than the other clusters. The different clusters were linked to different biological mechanisms, including epithelial-mesenchymal transition and immune-relevant pathways.

**Conclusion:** FAP is a promising biomarker to distinguish prognosis and is associated with the TME and ferroptosis in STAD.

## Introduction

Stomach adenocarcinoma (STAD) is a globally important disease. It represents the fifth most common cancer and the third leading cause of cancer death worldwide (Smyth, Nilsson, Grabsch, van Grieken, and Lordick, 2020). Early symptoms of STAD are not apparent. A lot of patients are diagnosed as terminal STAD along with lymph node infiltration and distant metastasis, resulting in a generally poor prognosis (Rawla and Barsouk, 2019). Further exploration of new biomarkers for STAD is important.

Fibroblast activation protein-alpha (FAP) was abundantly and stably expressed in cancer-associated fibroblasts (CAFs) in the cancer stroma. It is a transmembrane protein belonging to type II integral serine protease that possesses catalytic activity in the form of a dimer. Structurally, FAP was composed of extracellular domain, single transmembrane domain, and a cytoplasmic tail. It specifically cleaves the post-proline peptide bond while harboring both dipeptidyl peptidase and endopeptidase activities (Aertgeerts et al., 2005; Simkova, Busek, Sedo, and Konvalinka, 2020). FAP overexpression facilitates tumor progression and invasion by manipulating extracellular matrix remodeling, angiogenesis, intracellular signaling, epithelial-to-mesenchymal transition (EMT), and immunosuppression (Simkova et al., 2020). High expression of FAP has been implicated in several human malignancies, such as breast cancer (Meng et al., 2016), ovarian cancer (W. Yang et al., 2013), and STAD (Wen et al., 2017). Several studies have provided more specific conclusions regarding the effect of FAP on STAD. Wang et al. (R. F. Wang et al., 2013) documented the positive association of the overexpression of FAP in CAFs with STAD invasive depth, differentiation, Lauren classification, and TNM classification. Gao et al. (Gao et al., 2019) found that FAP promotes angiogenesis and results in a higher microvessel density in STAD. FAP can serve as an independent prognostic biomarker, promoting the deterioration of the tumor and leading to adverse clinical outcomes in STAD (Liu et al., 2018).

Ferroptosis was a novel discovered form of iron-dependent programmed cell death. Ferroptosis arises from the accumulation of lipid hydroperoxides and results in cytological changes. The characteristics and mechanisms of ferroptosis are distinct from typical cell death processes, including cell autophagy and apoptosis (Dixon et al., 2012). Ferroptosis was strongly linked to a variety of metabolic processes involving iron, amino acids, NADH, glutathione, and phospholipids (Liang, Zhang, Yang, and Dong, 2019). Ferroptosis participates in biological regulation and signal transduction pathways, contributing to tumor initiation and development (Stockwell et al., 2017; Shen et al., 2018; Stockwell and Jiang, 2019). There are a variety of genes that suppress ferroptosis in tumors, such as GPX4 (Friedmann Angeli et al., 2014) and SLC7A11 (Ma, Henson, Chen, and Gibson, 2016), whereas NOX1 (Dixon et al., 2012), ACSL4 (Yuan, Li, Zhang, Kang, and Tang, 2016), and LPCAT3 (Kagan et al., 2017) serve as drivers to promote ferroptosis. Recently, inducing ferroptosis to take place has turned into a potential way of therapy to effectively motivate cancer cell death, especially in cancers that are resistant to conventional therapy (Friedmann Angeli, Krysko, and Conrad, 2019). It has been reported that ferroptosis participated in the modulation of STAD (Hao et al., 2017; Niu, Zhang, Tong, Li, and Liu, 2019).

The relationship between FAP expression and ferroptosis in tumors is still unknown. In this study, we sought to integrate the genomic information of STAD samples to comprehensively correlate FAP expression with clinical characteristics and immune characteristics and further explore the association between FAP expression and ferroptosis. The elevated level of FAP expression was linked to a more advanced tumor stage in STAD. Four distinct ferroptosis-related clusters were identified based on the mRNA expression profiles of ferroptosis-related genes (FRGs). Surprisingly, the expression level of FAP was obviously lower in ferroptosis-related cluster C, which is related to prolonged survival.

## Methods and Materials

### Data Source and Differential Expression Analysis

The pan-cancer dataset from The Cancer Genome Atlas (TCGA), which comprises 33 types of normal and tumor samples, was downloaded from the UCSC Xena browser (http://xena.ucsc.edu/) (Goldman et al., 2020). The expression of FAP between tumor and normal samples was compared. Clinical information and somatic mutation data from TCGA-STAD were also retrieved from the UCSC Xena browser. RNA-seq data and clinical information of 300 stomach adenocarcinoma samples (Asian Cancer Research Group [ACRG] cohort) (Cristescu et al., 2015) were acquired from the Gene Expression Omnibus database (https://www.ncbi.nlm.nih.gov/geo/) for molecular subtype (Supplementary Table S1) distribution analysis.

### Pan-Cancer Analysis of FAP

Tumor mutational burden (TMB) is a marker that refers to the number of mutations in a cancer cell. The TMB of each tumor sample was calculated respectively. Microsatellite instability (MSI) can be used to reflect the appearance of a new microsatellite allele phenomenon compared to normal tissue. In a tumor, alterations in the length of a microsatellite are caused by insertion or deletion of a repeat unit (B. Zhang et al., 2020). We analyzed the association between FAP expression and MSI.

### Prognosis Analysis

Univariate survival analysis was performed in order to clarify the relationship between FAP expression and patient survival. Visualization was done through the “forest plot” package in R software. STAD samples were divided into high FAP expression group and low FAP expression groups according to the median value of FAP expression. Survival analysis was performed between subgroups.

### Correlation Analysis of FAP Expression With Clinicopathological and Molecular Characteristics

The correlation analysis of FAP expression with clinicopathological and molecular characteristics was performed. Statistical significance was tested using the Kruskal-Wallis rank sum test. To further explore the molecular characteristics of the FAP subgroups, the chi-square test was performed.

### CIBERSORT Estimation and Immune-Related Analysis

To identify the immune characteristics of STAD samples, the CIBERSORT algorithm was performed to assess the relative proportion of 22 kinds of immune cells and to estimate differences in the infiltration of 22 immune cell types between the two groups with high and low FAP expression. The results were presented in a landscape map. The ESTIMATE algorithm was used to assess the stromal score and immune score for each STAD sample. The Wilcoxon test was applied to clarify the differential expression of immune checkpoint genes between two groups with high and low FAP expression. Spearman’s relation between the expression of FAP and immune checkpoint genes was calculated for STAD. Using the Gene Expression Profiling Interactive Analysis database (http://gepia.cancer-pku.cn/) (Tang et al., 2017), we retrieved the correlations between FAP expression and PDCD1, CTLA4, and CD274 levels in STAD.

### Establishment of Competing Endogenous RNA Network in STAD

On account of the hypothesis that long non-coding RNA (lncRNA) indirectly modulates mRNA expression by competitively binding microRNA (miRNA), the ceRNA network was established. StarBase (https://starbase.sysu.edu.cn/index.php) (Li, Liu, Zhou, Qu, and Yang, 2014) was utilized to search the potential miRNAs targeted by FAP. Differential expression, survival, and correlation analyses of miRNAs were performed. Target miRNAs which were inversely linked to FAP were screened for the next analysis to predict the potential lncRNAs targeted by the selected miRNAs. The lncRNAs that were positively correlated with miRNAs were excluded. R software was used for differential expression analysis and survival analysis of lncRNAs. Finally, a lncRNA-miRNA-mRNA triple regulatory network was constructed by integrating the lncRNA-miRNA pairs with miRNA-mRNA pairs. Cytoscape version 3.8.2 was utilized to visualize the ceRNA regulatory networks.

### Ferroptosis-Based Consensus Clustering Analysis

FRGs were acquired from the FerrDb database (http://www.zhounan.org/ferrdb/legacy/index.html) (Zhou and Bao, 2020). The FRGs included 150 drivers and 159 suppressors. Correlation analysis of FAP with FRGs was performed. The expression of FRGs was compared between two groups with high and low FAP expression using the Wilcoxon test. Subsequently, based on the expression of 43 FRGs (Supplementary Table S2) related to FAP, the cluster analysis was conducted. The samples were divided into four subgroups using optimal k-means clustering (“kmeans” function in R). We conducted cluster analysis. The Kaplan-Meier method was employed to determine overall survival (OS) in the various clusters. Differences in FAP expression between the different ferroptosis-related phenotypes were identified. The chi-square test was carried out to investigate the ferroptosis-related phenotypes of the FAP subgroups.

### GSEA

To observe the interrelated biological functions and pathways of gene expression in tumors, the samples were classified into different ferroptosis-related subgroups according to ferroptosis-based consensus clustering analysis. We carried out GSEA (Mootha et al., 2003; Subramanian et al., 2005) in these groups. The “2.cp.kegg.v7.4.symbols” and “c5.all.v7.4.symbols” gene sets were downloaded from the Molecular Signatures Database (MSigDB) for GSEA.

### Statistical Analysis

The Wilcoxon test or the Kruskal-Wallis test was used to compare differences of FAP subtypes by the “ggpubr” package. The correlations between FAP and other factors, such as MSI and TMB, were tested by Spearman’s rank correlation coefficient. The correlation analyses between FAP and PDCD1, CTLA4, and CD274 were completed using web tool GEPIA. All survival analyses were performed by the “survival” and “survminer” packages. Survival curves were plotted using the Kaplan-Meier method. The module used a univariate Cox regression model to explore the prognosis and visualization was performed through the “forest plot” package. The chi-square test was performed to explore the molecular characteristics of the FAP subgroups. Correlation analysis of FAP with FRGs was visualized by “corrplot” package. We conducted cluster analysis applying the “ConsensusClusterPlus” R package. Statistical significance was set at *p* < 0.05. R version 4.1.1 was used for all analyses.

## Results

### FAP is Highly Expressed in Pan-Cancer

**FIGURE 1:**
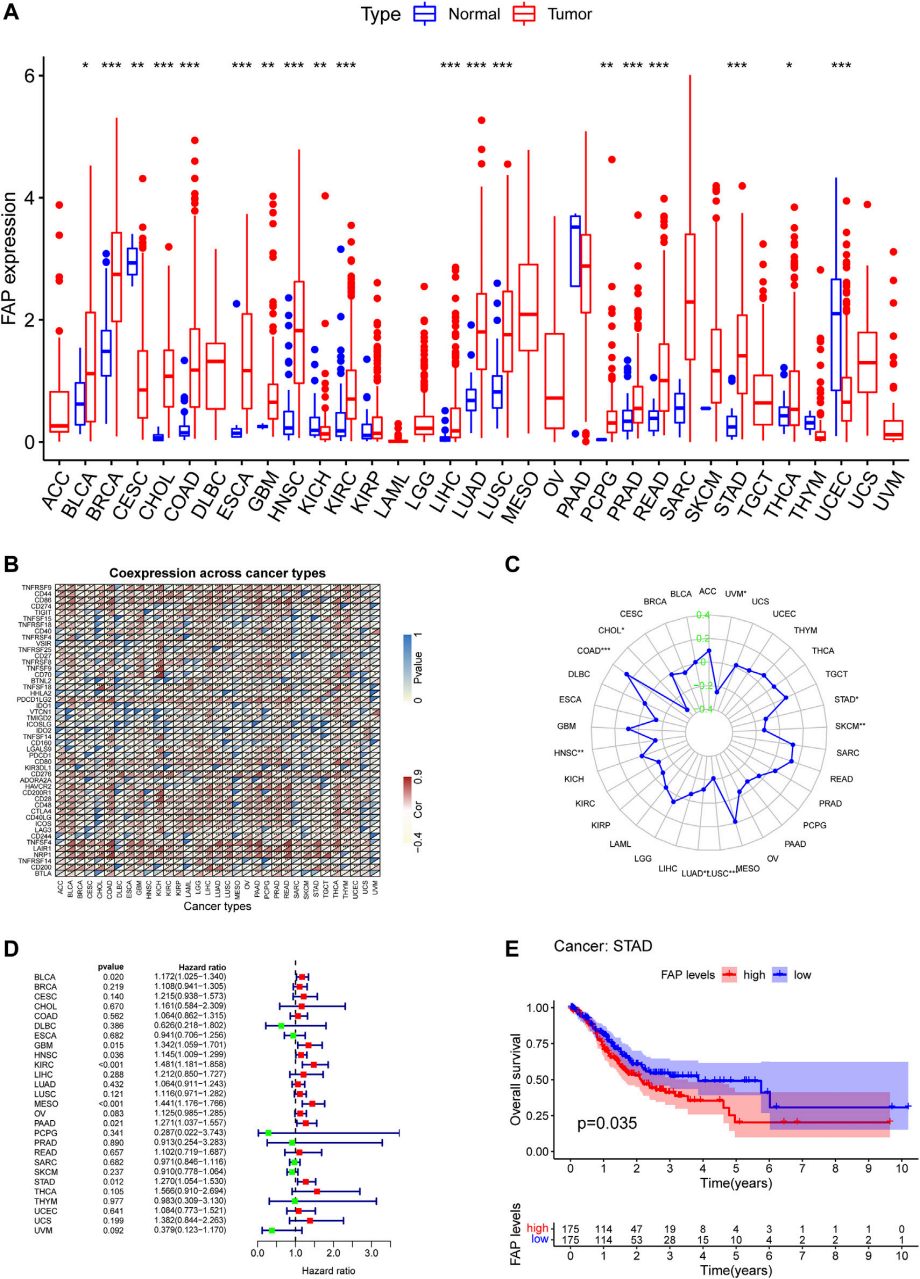
High FAP expression infers poor prognosis for STAD. **(A)** FAP expression levels in tumors compared to normal tissues in 33 cancers; *p < 0.05, **p < 0.01, ***p < 0.001. **(B)** Correlations between FAP expression and immune checkpoint gene expression in 33 cancers; *p < 0.05, **p < 0.01, ***p < 0.001. **(C)** Results of correlation analysis between FAP expression in pan-cancer and MSI described using Spearman’s rank correlation coefficient. **(D)** Univariate Cox analysis of FAP shown by a forest plot. A hazard ratio of >1 represented risk factors for survival and a hazard ratio of <1 represented protective factors for survival. The full version can be accessed in supplementary figure. **(E)** Kaplan–Meier plots of FAP in STAD. Patients were divided into high and low FAP expression groups based on the median FAP expression level.

Previous studies have documented the dysregulated expression of FAP in cancers (Meng et al., 2016; Wen et al., 2017; W.; Yang et al., 2013). We use TCGA data to analyzed the expression levels of FAP by comparing it in 33 types of cancers vs. adjacent samples. By examining the differential expression between cancer and normal samples, we observed that the expression level of FAP was generally lower in normal samples compared to tumors across these cancer types (Figure 1A).

The correlation between FAP and immune checkpoint genes could be evaluated based on the expression of more than 40 immune checkpoint genes generally found in different kinds of cancers. FAP expression was positively related to the expression levels of immune checkpoint genes in different kinds of cancers, including colon adenocarcinoma (COAD), thyroid cancer, STAD, and liver hepatocellular carcinoma (LIHC), indicating that FAP may have a potential effect on modulating tumor immune pattern in some cancers by modulating the immune checkpoint genes expression levels (Figure 1B).

MSI is defined as the appearance of a new microsatellite allele at a microsatellite locus in a cancer compared to normal tissue because of the insertion or deletion of a repeat unit (Krieger, Pearson, Bell, Doherty, and Robbins, 2020). The association between FAP expression and MSI was analyzed using Spearman’s relation. FAP expression was positively linked to MSI in colon adenocarcinoma and negatively related to cholangiocarcinoma, lung adenocarcinoma, uveal melanoma, STAD, lung squamous cell carcinoma (LUSC), skin cutaneous melanoma, and head and neck squamous cell carcinoma (HNSC) (Figure 1C).

TMB refers to the total number of somatic mutations taking place at an average of per million bases in the coding regions of the cancer cell genome. The mutation types comprise different forms of mutations, such as small insertions/deletions (indels) and single nucleotide variations. TMB is a marker that reflects the number of mutations in cancer cells (Krieger et al., 2020). As shown in Supplementary Figure S1A, Spearman’s relation was conducted to analyze the association between FAP expression and TMB for each cancer type separately. Notably, FAP expression was positively related to TMB in adenoid cystic carcinoma (ACC), thymoma, sarcoma, prostate adenocarcinoma, ovarian cancer (OV), acute myeloid leukemia, kidney chromophobe (KICH), and COAD, and negatively correlated with pancreatic adenocarcinoma (PAAD), LUSC, LIHC, HNSC, and kidney renal papillary cell carcinoma (KIRP).

Univariate survival analysis was employed to calculate the correlation between FAP expression and overall survival for 33 kinds of tumors in TCGA. As displayed in Figure 1D and Supplementary Figure S1B, the forest plots among the 33 types of cancers demonstrated that FAP could significantly influence the OS of ACC (hazard ratio [HR] = 2.187, 95% CI 1.526–3.134, *p* < 0.001), bladder urothelial carcinoma (BLCA; HR = 1.172, 95% CI 1.025–1.340, *P* = 0.020), glioblastoma (GBM; HR = 1.342, 95% CI 1.059–1.701, *P* = 0.015), HNSC (HR = 1.145, 95% CI 1.009–1.299, *P* = 0.036), KICH (HR = 4.118, 95% CI 1.389–12.204, *P* = 0.011), kidney renal clear cell carcinoma (KIRC; HR = 1.481, 95% CI 1.181–1.858, *p* < 0.001), KIRP (HR = 4.638, 95% CI 2.955–7.281, *p* < 0.001), low-grade glioma (LGG; HR = 2.366, 95% CI 1.621–3.454, *p* < 0.001), mesothelioma (MESO; HR = 1.441, 95% CI 1.176–1.766, *p* < 0.001), PAAD (HR = 1.271, 95% CI 1.037–1.557, *P* = 0.021), and STAD (HR = 1.270, 95% CI 1.054–1.530, *P* = 0.013) patients. The findings indicated that FAP was linked to poor patient prognosis. Furthermore, Kaplan–Meier curves of FAP in STAD revealed better survival of patients with low FAP expression (Figure 1E), suggesting that FAP may be a potential prognostic indicator in STAD.

### Relationship Between Expression Level of FAP and Other Clinical Characteristics

**FIGURE 2:**
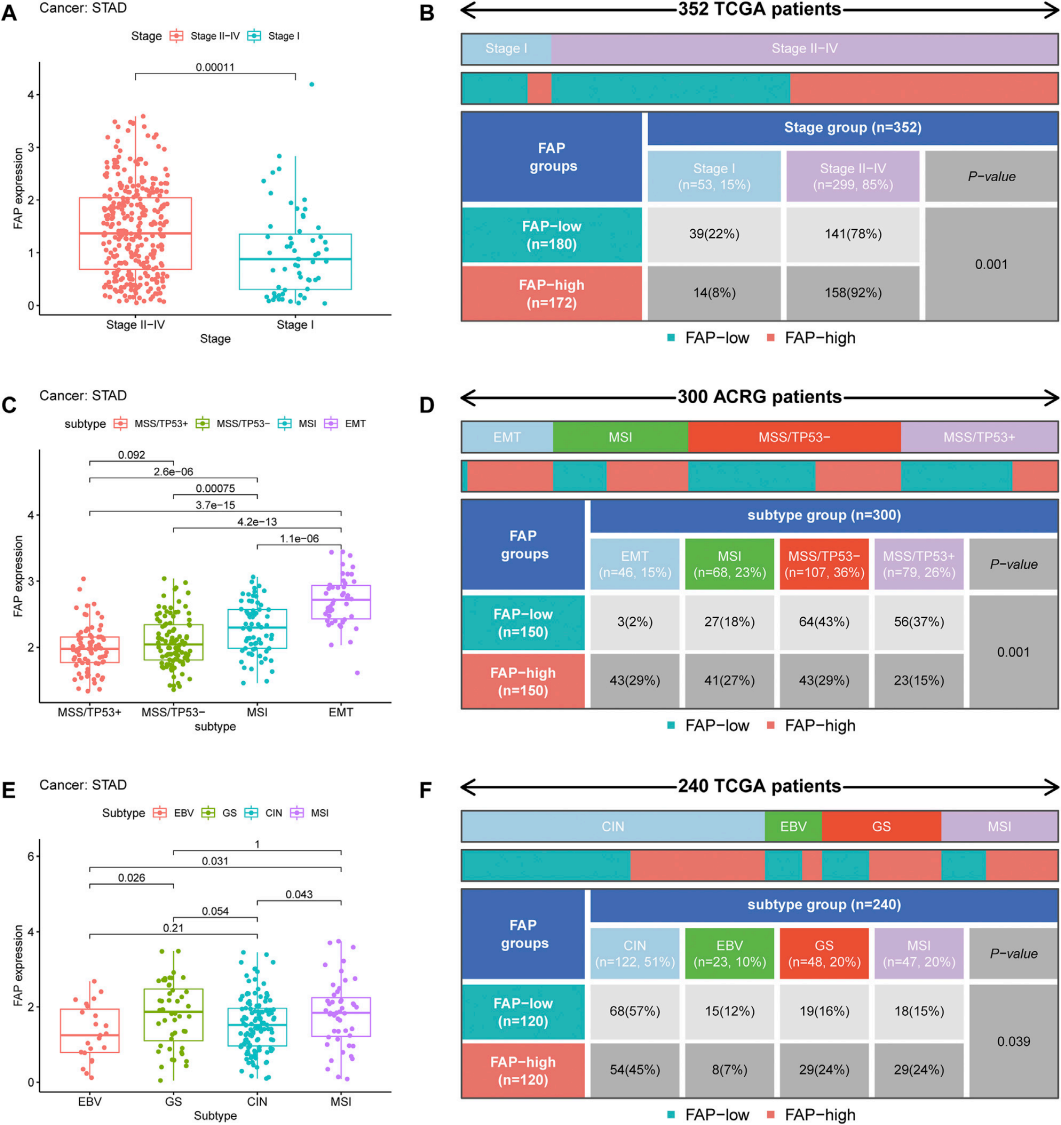
Clinical correlation of FAP. **(A)** FAP expression level in different STAD stages. **(B)** Heat map and table showing the distribution of STAD stages among the different FAP expression levels. **(C)** FAP expression levels in different ACRG molecular subtypes. **(D)** Heat map and table showing the distribution of ACRG molecular subtypes among the different FAP expression levels. **(E)** FAP expression level in different TCGA molecular subtypes. **(F)** Heat map and table showing the distribution of TCGA molecular subtypes among the different FAP expression levels.

Analysis of the relationship between FAP expression and clinical characteristics displayed that the elevated level of FAP expression was linked to a more advanced cancer stage in STAD (Figure 2A and Supplementary Figure S1C). In addition, as shown in Figure 2B and Supplementary Figure S1D, more advanced tumor stages were distributed in the FAP-high subgroup (*P* = 0.001; *P* = 0.005).

To further explore the characteristics of FAP expression in various clinical features and biological functions, we concentrated on the ACRG cohort of 300 stomach adenocarcinoma samples, which contains extensive clinical information. Large genomic profiling studies of STAD in the ACRG cohort have consistently reported four distinct molecular subtypes: MSS/TP53+, MSI, EMT, and MSS/TP53-. We noticed that FAP expression was significantly higher in the EMT subtype than in MSI, MSS/TP53-, and MSS/TP53+ (*p* < 0.05, Figure 2C). Then we concentrated on different subtypes in the FAP subgroups. The FAP-low subgroup consisted of 2% EMT samples, 18% MSI samples, 43% MSS/TP53- samples, and 37% MSS/TP53 + samples. The FAP-high subgroup comprised 29% EMT samples, 27% MSI samples, 29% MSS/TP53- samples, and 15% MSS/TP53 + samples (Figure 2D). There were more EMT samples in FAP-high subgroup compared to FAP-low subgroup (*P* = 0.001, chi-square test). In STAD, the EMT molecular subtype was obviously associated with poorer prognosis, whereas MSI was correlated with better survival. Therefore, tumors featured by FAPs were importantly related to stromal activation, high degree of malignancy, and rapid development.

A comprehensive molecular landscape has been established for STAD using TCGA data, which classifies STAD into the following molecular subtypes: genome stable (GS), MSI, Epstein–Barr virus (EBV) infection, and chromosomal instability (CIN). We investigated differences in FAP expression between these molecular subtypes. An increase in FAP expression was observed in the GS subtype (Figure 2E). As shown in Figure 2F, there were more GS samples in the FAP-high group compared to the FAP-low group (*P* = 0.039, chi-squared test). The data suggested that FAP expression can predict the stages of STAD and patient prognosis.

### Immune Characteristics of Different FAP Subgroups

**FIGURE 3:**
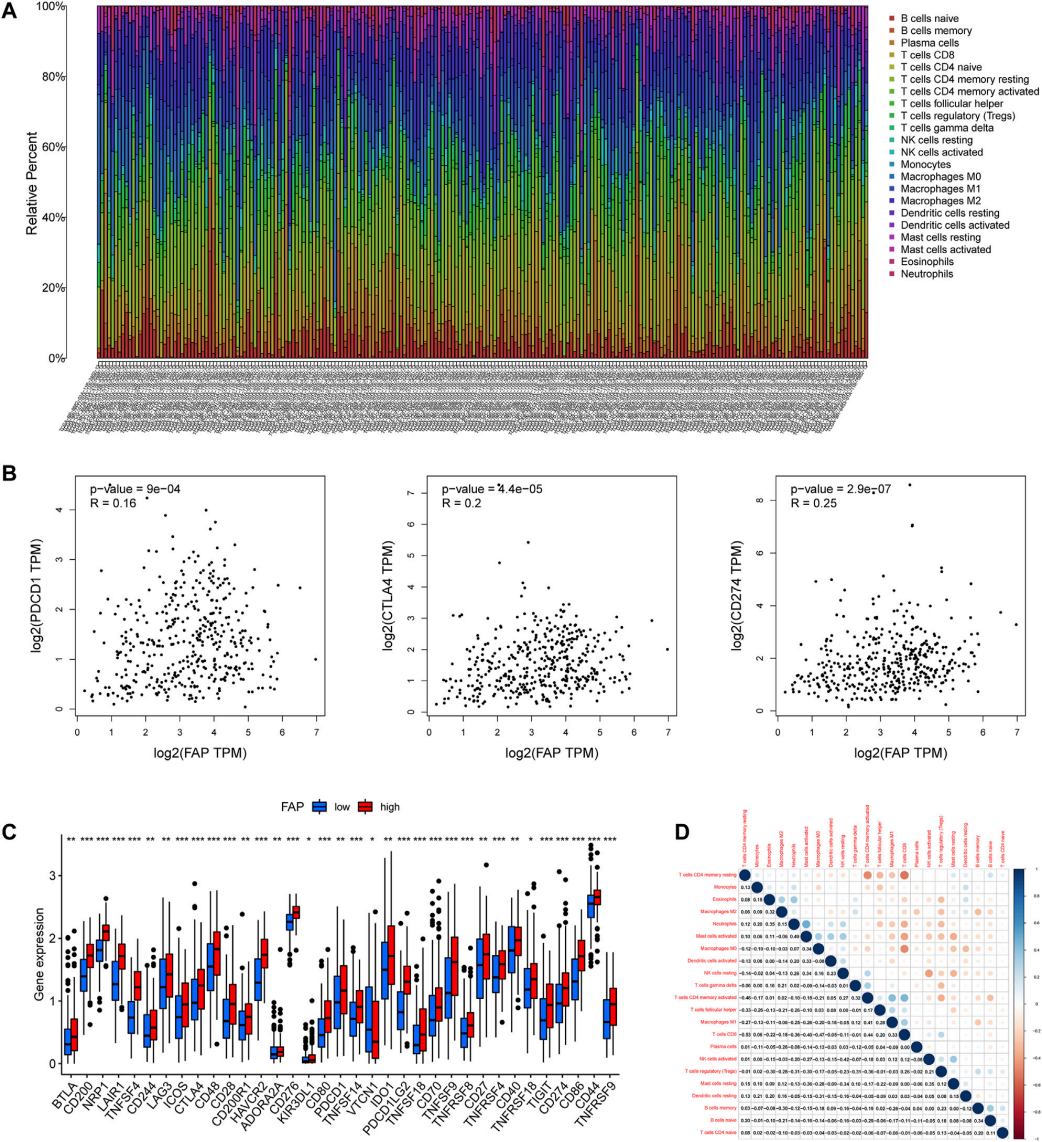
Landscape of the TME in STAD and characteristics of different FAP expression levels. **(A)** Proportions of TME cells among patients with STAD in the TCGA cohort. **(B)** Correlation plots showing correlations between FAP expression and expression of PDCD1, CTLA4, and CD274 in STAD patients. *R* = linear correlation coefficient. *P* = *p*-value of linear correlation. **(C)** Expression levels of immune checkpoint genes in FAP-high and FAP-low groups; *p < 0.05, **p < 0.01, ***p < 0.001. **(D)** Correlation analysis was used to assess relationships among immune cells.

To illustrate the proportion of immune landscapes in STAD, the CIBERSORT deconvolution algorithm using support vector regression was applied to determine the immune cell type in cancers and explore various distribution of immune cells among the different patients. The characteristics of the immune landscape are shown in Figure 3A.

To evaluate the composition of immune cells between different FAP subgroups, the Wilcoxon test was conducted to analyze the component of immune cells in the two FAP subgroups. There are more M0 macrophages, and M2 macrophages distributed in the FAP-high subgroup, whereas more naïve B cells, memory B cells, and plasma cells were distributed in the FAP-low subgroup (Supplementary Figure S1E). We also investigated correlations between FAP and immunogenic cell death (ICD) in 33 cancers. As shown in Supplementary Figure S1F, FAP expression was positively related to the expression of ICD in COAD, PAAD, and STAD. The R package for immune and stromal scoring of individual tumor samples revealed that FAP expression was positively correlated with immune score as well as stromal score (Supplementary Figure S2A). Immune infiltration analysis indicated that FAP expression was positively linked to the infiltration of M1 macrophages, M2 macrophages, eosinophils, monocytes, resting mast cells, and neutrophils. Two types of immune cells had a negative relationship with FAP expression: naïve B cells and CD4^+^ resting memory T cells (Supplementary Figure S2B).

We further studied whether FAP affected cancer response to immune therapy. First, we sought to understand the correlation between FAP and immune checkpoint gene expression in patients, including Programmed Cell Death Protein 1 (PDCD1), cluster of differentiation 274 (CD274, also termed PD-L1), and Cytotoxic T-Lymphocyte Associated Protein 4 (CTLA4). FAP displayed a moderately positive correlation with PDCD1, CTLA4, and CD274 (Figure 3B).

Patients with elevated level of FAP expression exhibited obviously elevated levels expression of PDCD1, CTLA4, and CD274 (Figure 3C), suggesting a possible response to immunotherapy of anti-PD-1/L1. Besides, low to moderate correlations were observed in various immunocyte subpopulations (Figure 3D).

### Construction of ceRNA Network

**FIGURE 4:**
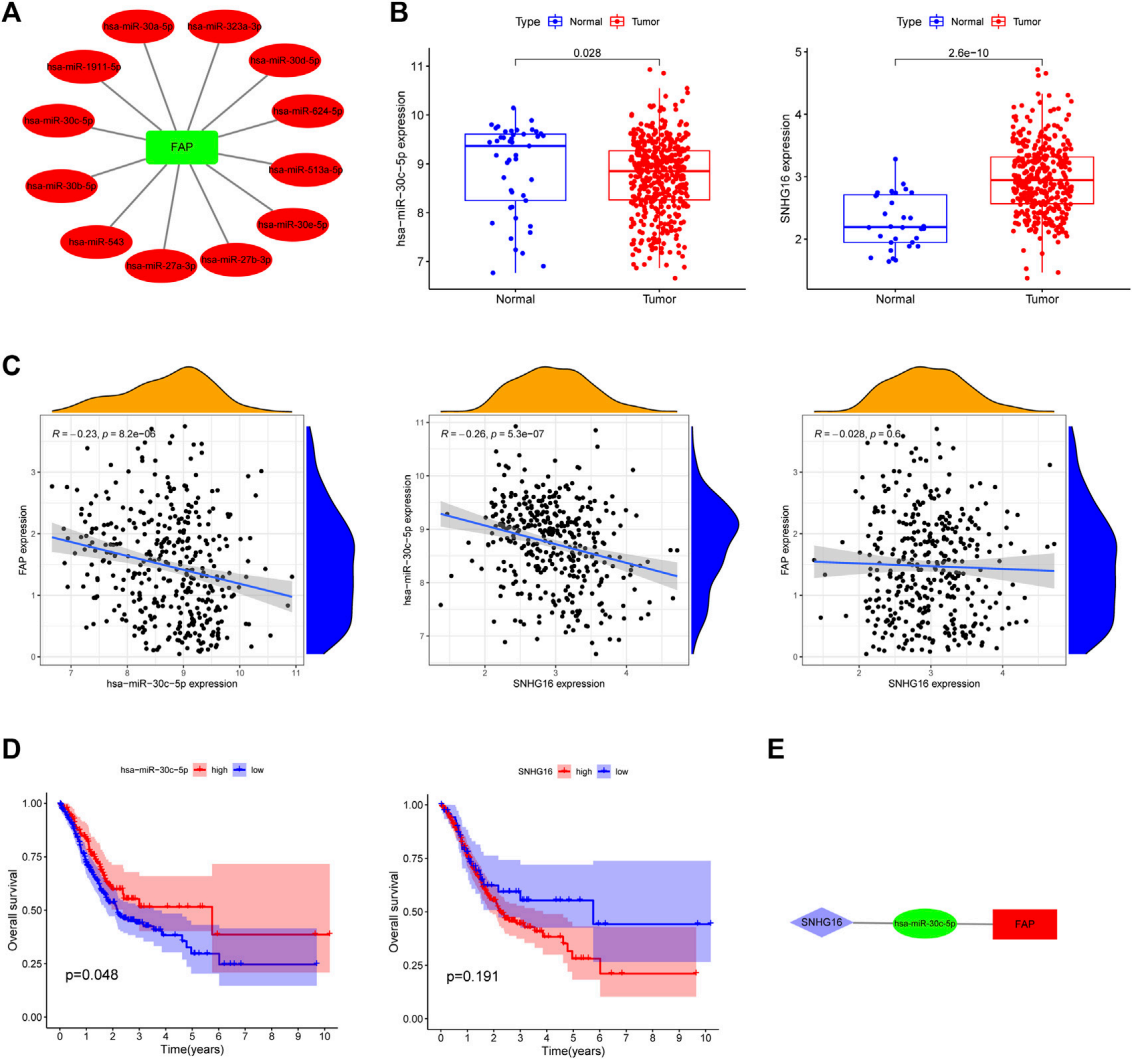
Identification of a FAP regulatory network. **(A)** A correlation network between FAP and miRNAs. **(B)** Expression of hsa-miR-30c-5p and SNHG16 in TCGA. **(C)** Association between FAP, SNHG16 and hsa-miR-30c-5p evaluated using the TCGA database. **(D)** Overall survival of hsa-miR-30c-5p and SNHG16 assessed using Kaplan–Meier Plotter. **(E)** A triple regulatory network in STAD.

The miRNAs upstream of FAP were searched using the StarBase database. A total of 12 upstream miRNAs capable of interacting with FAP were identified. The mRNA-miRNA regulatory networks for upregulated mRNAs, comprising 12 miRNA-mRNA pairs, were established and visualized by Cytoscape (Figure 4A).

We hunted for miRNAs which differentially expressed between cancer and normal patients in STAD. The following miRNAs were identified: hsa-miRNA-30c-5p (Figure 4B), hsa-miRNA-30b-5p, hsa-miRNA-30d-5p, and hsa-miRNA-624-5p (Supplementary Figure S3A). The correlation between the four miRNAs and FAP in STAD (Figure 4C and Supplementary Figure S3B) was analyzed. Only one inversely correlated miRNA-mRNA pair (hsa-miRNA-30c-5p/FAP) was evident. As shown in Figure 4D, hsa-miRNA-30c-5p was a prognostic biomarker according to the OS analysis of patients with STAD (*P* = 0.048). Overall survival analyses of other miRNAs were shown in Supplementary Figure S3C.

We then searched for potential upstream lncRNAs of hsa-miRNA-30c-5p using StarBase. Significantly differentially expressed lncRNAs were also identified (Figure 4B). SNHG16 expression is positively correlated with hsa-miRNA-30c-5p expression (Figure 4C). According to the above information, a lncRNA-miRNA-mRNA network was constructed (SNHG16/hsa-miRNA-30c-5p/FAP, Figure 4E). No significant association was shown between FAP and SNHG16 expression in STAD (Figure 4C). No significantly prolonged survival of SNHG16 was observed (*P* = 0.191, Figure 4D).

### Ferroptosis-Related Clusters Mediated by 43 FRGs

**FIGURE 5:**
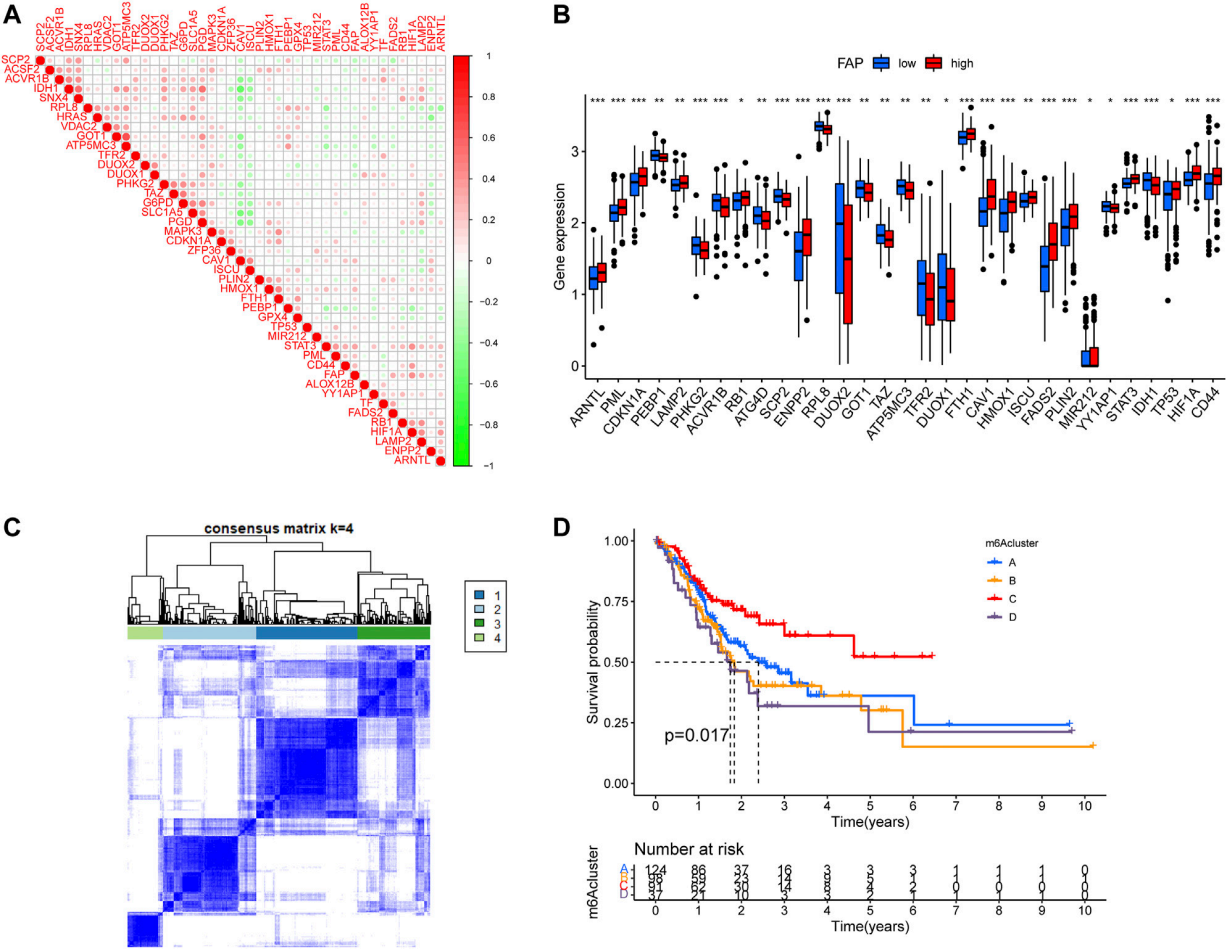
Consensus clustering of ferroptosis-related genes. **(A)** Correlations between FAP and known FRGs in the TCGA-STAD cohort. **(B)** Expression levels of FRGs in FAP-high and FAP-low groups; *p < 0.05, **p < 0.01, ***p < 0.001. **(C)** Consensus clustering matrix for *k* = 4. **(D)** Kaplan–Meier survival analysis for patients in the four ferroptosis-related clusters.

A total of 43 FRGs, including 19 suppressors and 24 drivers, were identified. To better understand the characteristics of FAP, we evaluated the association between FAP expression and known FRGs (Figure 5A). The FRGs ARNTL, PML, CDKN1A, LAMP2, RB1, ENPP2, FTH1, CAV1, HMOX1, ISCU, FADS2, PLIN2, TP53, HIF1A, and CD44 were significantly upregulated in FAP-high subgroup compared to those in FAP-low subgroup. The PEBP1, PHKG2, ACVR1B, ATG4B, SCP2, RPL8, DUOX2, TAZ, TFR2, IDH1, and DUOX1 genes were markedly downregulated (Figure 5B). Expression levels of drivers and suppressors in FAP-high as well as FAP-low subgroups were exhibited in Supplementary Figure S4A,B, respectively.

The unsupervised clustering algorithm revealed four distinct ferroptosis-related phenotypes (clusters A–D) (Figure 5C). Prognostic analysis demonstrated that cluster C was closely correlated with a survival advantage. Clusters A, B, and D were related to worse prognosis (*P* = 0.017, Figure 5D).

### Characteristics of Ferroptosis-Related Clusters in FAP Subgroups and GSEA

**FIGURE 6:**
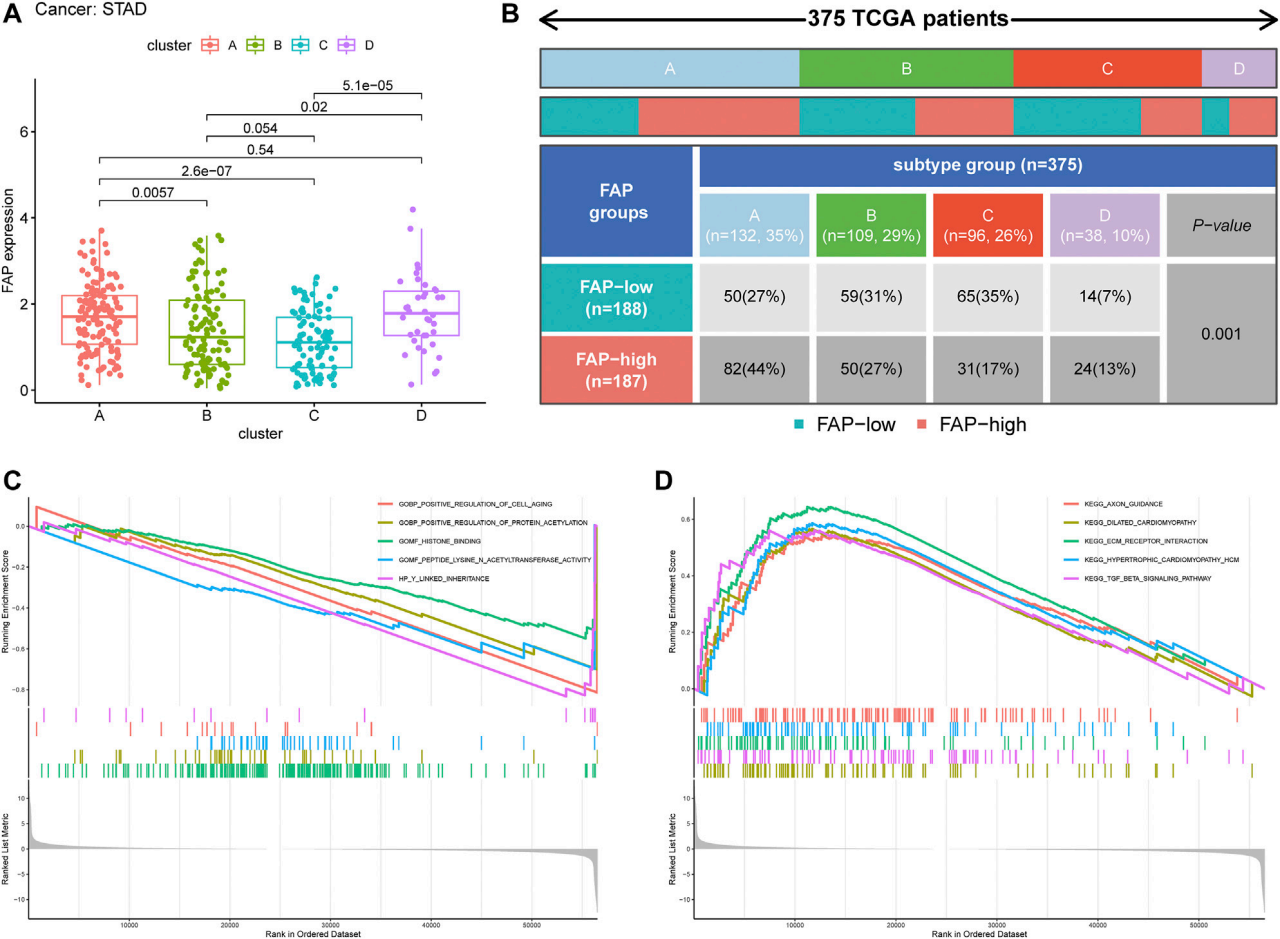
Clinical correlation of different subtypes. **(A)** Distribution of FAP expression across the four STAD subtypes. **(B)** Heat map and table showing the distribution of FAP expression levels among the different subtypes. **(C and D)** The results of GSEA based on GO database **(C)** and KEGG **(D)** in subtype C.

We evaluated the differences in FAP expression between the four ferroptosis-related clusters. The expression level of FAP was remarkably lower in ferroptosis-related cluster C than in ferroptosis-related clusters A, B, and D (*p* < 0.05, Figure 6A). More ferroptosis-related cluster C subtypes were evident in the FAP-low subgroup, and more ferroptosis-related cluster A and ferroptosis-related cluster D subtypes in the FAP-high subgroup (Figure 6B; *P* = 0.001, chi-square test).

To further evaluate the possible biological functions of each ferroptosis-related cluster, GSEA was conducted to determine the gene sets enriched in different clusters (Supplementary Tables S3, S4). The gene sets of the ferroptosis-related cluster C samples were enriched in the extracellular matrix (ECM) receptor interaction and transforming growth factor-beta (TGF-β) signaling pathway (Figures 6C,D). Gene sets of clusters A and B were mainly involved in olfactory activity (Supplementary Figures S5A,B). Cluster D was particularly enriched in leukocyte migration, cytokine-cytokine receptor interactions, and chemokine signaling pathways (Supplementary Figure S5A,B).

## Discussion

Most patients with STAD tend to be diagnosed as terminal cancers (Wagner et al., 2002). Very few of these benefits from the development of targeted therapy (Brar and Shah, 2019; Ilson, 2019; B. C.; Wang, Zhang, Fu, and Wang, 2019). And then prognosis of STAD remains poor. Thus, discovery of novel biomarkers to detect and predict the survival of patients with STAD is urgent.

Previous studies have shown that CAF upregulation of FAP drives cancer progression and invasion by affecting ECM remodeling, immunosuppression, EMT, angiogenesis, and intracellular signaling (Krepela et al., 2021). However, little is known about the molecular character of FAP in STAD and its capability to modulate the TME. In the current study, we attempted to explore the relationship between FAP and STAD to enhance our comprehension of antitumor responses and inform more valid strategies for patients.

We observed that FAP was highly upregulated in most of cancers, including STAD. To further probe the function of FAP in tumor prognosis prediction, we conducted a series of pan-cancer studies including TCGA cohort patients and observed that high expression of FAP predicted adverse prognosis compared to low FAP expression at the pan-cancer level. Furthermore, to investigate the relation between FAP and STAD, the median value of FAP expression was used as the division basis to classify TCGA patients into two groups with high and low expression of FAP. Elevated level of FAP expression was more likely to be correlated with a worse survival and a more advanced tumor stage. It is known that FAP is a vital marker of CAFs and contributes to tumor proliferation and metastasis, which may be due to its regulation of the structure and the extracellular matrix component (Santos, Jung, Aziz, Kissil, and Puré, 2009; Lee et al., 2011; Mazur, Holthoff, Vadali, Kelly, and Post, 2016), involving in EMT and angiogenesis (Huang, Wang, and Kelly, 2004; Koczorowska et al., 2016; Wu et al., 2020), which might account for a larger proportion of advanced-stage patients in FAP-high group.

In STAD, the EMT molecular subtype was correlated with worse clinical outcomes, while MSI was correlated with better prognosis (Cristescu et al., 2015). In the ACRG cohort, patients with EMT subtype were linked to higher expression of FAP. In accordance with the above results, the majority of patients with EMT subtypes were divided into the FAP-high subgroup, and only a few EMT subtypes were in the FAP-low subgroup, again indicating that high FAP expression tended to be related to adverse clinical outcomes. Jia et al. (Jia et al., 2018) demonstrate that FAP could reduce both E-cadherin and *β*-catenin expression through the sonic hedgehog pathway in tumor, and promote epithelial-mesenchymal transition, finally generating distant metastasis of tumor. Liu et al. (Liu et al., 2018) revealed that FAP could drive the development of GC via EMT mechanism through Wnt/β-catenin pathway. The above discussed mechanism of FAP might be obligated to our result that a majority of patients with EMT subtypes were divided into the FAP-high subgroup. Previous studies have demonstrated that EBV-infected GC patients have been reported to react to anti-PD-1/L1 antibodies, in spite of the lower MSI or TMB (Kim et al., 2018; Panda et al., 2018). In this study, patients with EBV-positive subtype were related to lower FAP expression than MSI, GS, and CIN subtype patients. These findings imply that FAP could be an efficient marker for prediction of immunological therapy effectiveness in patients with STAD.

It has been revealed that infiltration of immune cell can influence patient survival in previous studies (Yang et al., 2020; Zhang et al., 2021). In the current study, FAP expression was positively linkded to the infiltration levels of eosinophils, monocytes, M1 macrophages, resting mast cells, M2 macrophages, and neutrophils in STAD, while infiltration levels of several immune cells, including naïve B cells and CD4^+^ resting memory T cells, were inversely linked to the expression of FAP. Various studies have revealed that M2 macrophage infiltration contributes to tumor progression and the development of an invasive phenotype, which eventually leads to a poor prognosis in breast, bladder, ovarian, and gastric cancers (Josephs, Bax, and Karagiannis, 2015; Ruffell and Coussens, 2015; Fridman, Zitvogel, Sautès-Fridman, and Kroemer, 2017; Gambardella et al., 2020). The findings in the current study imply that STAD with high expression of FAP can lead to immune escape by promoting immunosuppressive cells, such as M2 macrophages, resulting in the development of STAD. Conversely, dense infiltration of T cells tends to result in a favorable outcome (Bindea et al., 2013; Gentles et al., 2015; Fridman et al., 2017). Immune checkpoint inhibitors were utilized to block inhibitory signaling and directly irritate the cytotoxic T lymphocytes activation to achieve antitumor effects (López-Soto, Gonzalez, and Folgueras, 2017; Jiang et al., 2018), promoting the eliminating capability of T cells against tumor cells. We also probed the differences between FAP and known predictive biomarkers for immunotherapy, including PDCD1, CTLA4, and CD274. Patients with high level of FAP displayed obviously elevated level of PDCD1 and CD274 expressions, and positive relationship between FAP expression and PDCD1, CTLA4, and CD274. The findings may indicate the participation of FAP in the development and application of immunotherapy in STAD. The collective findings suggest that FAP is likely to have an influence on the modulation of the TME and progression in STAD.

ceRNA is a gene expression regulation mechanism for post-transcriptional regulation presented by Salmena et al. (Salmena, Poliseno, Tay, Kats, and Pandolfi, 2011). The ceRNA theory states that endogenous RNA molecules can competitively bind to miRNAs due to the miRNA target sites, thus modulating miRNA target genes expressions indirectly (Salmena et al., 2011). A total of 12 correlated miRNA-mRNA pairs were identified. However, 11 miRNA-mRNA pairs were excluded which were not in accordance with the expected association with survival and FAP according to the ceRNA hypothesis. The results show that there is a correlation between FAP and miRNAs but that does not mean that miRNAs affect FAP expression. Here, we looked for upstream miRNAs and lncRNAs based on FAP and established the ceRNA network (SNHG16/hsa-miRNA-30c-5p/FAP) of STAD. Previous studies have indicated that SNHG16 promotes tumor development through EMT and predicts poor survival outcomes in several cancers (Cao, Xu, and Yue, 2018; Bu, Guo, Xu, Luo, and Liu, 2021). However, in the present study, no significant relationship was shown between FAP and SNHG16 in STAD. Furthermore, we did not find significantly prolonged survival of SNHG16 in STAD. The association between FAP and antitumor activity in STAD may not be fully explained by the ceRNA network. Other mechanisms warrant further investigation.

Ferroptosis is a new form of programmed cell death arising from the deposition of lipid peroxides in an iron-dependent manner. The character of other CAFs in regulating ferroptosis of tumor cells and promoting acquired chemoresistance in GC has been reported previously (H. Zhang et al., 2020). However, the interaction between FAP and ferroptosis remains unexplored. To further validate the regulatory mechanism of FAP, we first focused on the association of FRGs with FAP. FAP is inversely related to some drivers, which are genes that promote ferroptosis, such as ATP5MC3 and GOT1. However, it was positively correlated with suppressors, including GPX4 and FTH1, which prevent ferroptosis. GPX4 is a key gene to regulate ferroptosis and can promote tumor development (W. S. Yang et al., 2014). The influence of GPX4 on cancer prognosis agreed with that of FAP, indicating a significant relationship between FAP and ferroptosis. We then conducted unsupervised clustering analyses based on 43 FRGs, which revealed four distinct ferroptosis-related phenotypes. These four ferroptosis-related clusters had significantly distinct prognoses and clinical characteristics. Cluster C displayed a better prognosis than the other three clusters. In addition, patients in cluster C showed the lowest FAP expression compared with the other three clusters. To further investigate the biological behaviors of these four ferroptosis-related clusters, KEGG and GO analyses of the various clusters via GSEA revealed different biological functions in different ferroptosis-related clusters. Cluster C was involved in the ECM receptor interaction and TGF-β signaling pathway, which are significantly correlated with EMT. Genes from immune-relevant pathways, including chemokine signaling pathways, cytokine-cytokine receptor interactions, and leukocyte migration, tended to be enriched in cluster D. Olfactory activity was enriched in clusters A and B. These findings suggest that the different biological functions between the ferroptosis-related clusters may be the factors leading to diverse prognoses and clinical characterizations in STAD. The prognostic values of FAP have been confirmed in this study. The research provides valuable insight on the role of FAP in TME and reveals association between FAP and ferroptosis. However, the mechanism of interaction between FAP and ferroptosis in STAD remains unclear. More studies and experiments on this topic need to be undertaken before the relationship between FAP and ferroptosis is clearly understood.

In summary, we have identified FAP as a significant biomarker of STAD. The difference in FAP expression could not be ignored because of the heterogeneity of prognosis in STAD. Overexpression of FAP contributed to adverse survival in patients with STAD, particularly in the advanced clinical stage. Further analysis revealed a close relationship of FAP displays with ferroptosis. FAP may be non-negligible in STAD.

## Data Availability

Publicly available datasets were analyzed in this study. This data can be found here: The UCSC Xena browser (http://xena.ucsc.edu/) and the Gene Expression Omnibus database (https://www.ncbi.nlm.nih.gov/geo/).

## Abbreviations

FAP, Fibroblast activation protein-α; CAFs, Cancer-associated fibroblasts; STAD, Stomach adenocarcinoma; TCGA, The Cancer Genome Atlas; FRGs, Ferroptosis-related genes; ICB, Immunological checkpoint blockade; TME, Tumor microenvironment; ceRNA, competing endogenous RNA; lncRNA, long non-coding RNA; miRNA, microRNA; mRNA, messenger RNA; GO, gene ontology; GSEA, gene set enrichment analysis; KEGG, Kyoto encyclopedia of genes and genomes; TMB, Tumor mutational burden; MSI, Microsatellite instability; GS, Genome stable; CIN, Chromosomal instability; EBV, Epstein-Barr virus; CTLA-4, T-lymphocyte antigen-4; CD274, cluster of differentiation 274; PD-L1, Programmed cell death 1 ligand 1; PD-1, programmed cell death protein-1; EMT, epithelial-mesenchymal transition; CI, Confidence interval; HR, hazard ratio.
