# Supplementary figures and images for: Fibroblast Activation Protein-Alpha is a Prognostic Biomarker Associated With Ferroptosis in Stomach Adenocarcinoma
Source: Front Cell Dev Biol. 2022 Mar 14;10:859999. doi: 10.3389/fcell.2022.859999 (PMC8963861; doi:10.3389/fcell.2022.859999)

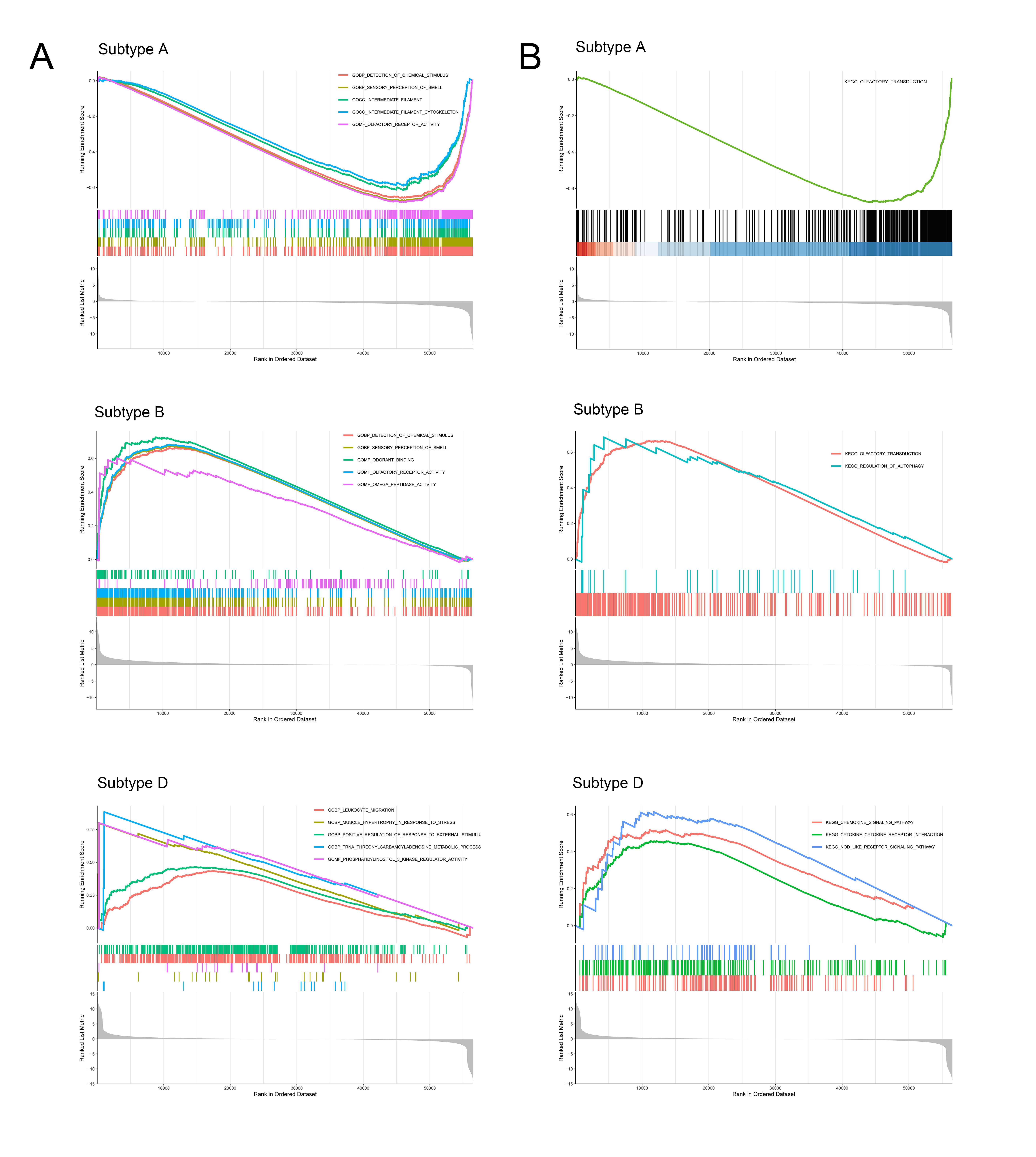

Supplement: Supplementary file 3 [file Image5.TIF]
